# Using Electrostatic Mapping to Understand PANI-MWCNTs’ NH_3_ Sensing

**DOI:** 10.3390/s26072169

**Published:** 2026-03-31

**Authors:** Alessia Famengo, Carmen Marinela Mihailescu, Mihaela Savin, Alexandru Grigoroiu, Carmen Moldovan, Maria Losurdo

**Affiliations:** 1 Institute of Condensed Matter Chemistry and Technologies for Energy—National Research Council of Italy (CNR-ICMATE), 35127 Padua, Italy; alessia.famengo@cnr.it; 2 National Institute for Research and Development in Microtechnologies (IMT), 077190 Bucharest, Romania; carmen.mihailescu@imt.ro (C.M.M.); mihaela.savin@imt.ro (M.S.); alexandru.grigoroiu@imt.ro (A.G.); carmen.moldovan@imt.ro (C.M.)

**Keywords:** NH_3_ sensor, PANI, MWCNTs, electrostatic force microscopy, charge delocalization, electrochemical impedance spectroscopy

## Abstract

This study investigates the electrostatic and electrochemical behavior of polyaniline (PANI) and its composite with amine-functionalized multi-walled carbon nanotubes (PANI/MWCNT–NH_2_) to elucidate the mechanisms governing ammonia (NH_3_) sensing. High-resolution atomic force microscopy (AFM) coupled with electrostatic force microscopy (EFM) demonstrates that pristine PANI forms granular macroaggregates with localized charge distribution, whereas MWCNT incorporation promotes worm-like percolative networks that enhance charge delocalization and conductivity. Electrochemical characterization by cyclic voltammetry (CV) and electrochemical impedance spectroscopy (EIS) corroborates these nanoscale observations, revealing significantly improved interfacial electron transfer kinetics in the composite. Upon exposure to NH_3_, pristine PANI undergoes rapid de-doping and nonlinear signal suppression, while the composite exhibits a more progressive electrochemical modulation. Overall, the results demonstrate that NH_3_ sensing in PANI-based films is governed not solely by electroactive material content but by the interplay between nanoscale morphology, electrostatic heterogeneity, and charge transport topology. The nanotube-mediated formation of delocalized and percolative conductive pathways provides structural and electrochemical robustness, enabling tunable, high-sensitivity operation suitable for next-generation, low-power ammonia sensing platforms.

## 1. Introduction

Ammonia (NH_3_) is a toxic and corrosive gas widely released from agricultural, industrial, and biomedical sources, and has been classified as a critical air pollutant due to its harmful effects on human health even at low concentrations [1]. Reliable, low-power, room-temperature sensing of NH_3_ is therefore essential for environmental monitoring, industrial safety, and for emerging non-invasive diagnostic applications such as breath analysis for liver and kidney dysfunction [2,3,4,5,6].

Conducting polymers, such as polyaniline (PANI), have been extensively investigated as active sensing layers for NH_3_ detection because of their reversible protonation/deprotonation behavior, facile electrochemical synthesis, and chemical stability under ambient conditions [7,8,9,10,11]. The sensing performance of PANI thin films strongly depends on their nanoscale morphology and local charge distribution, which are influenced by synthesis parameters and the presence of nanofillers [12,13,14,15,16,17].

**Figure 1:**
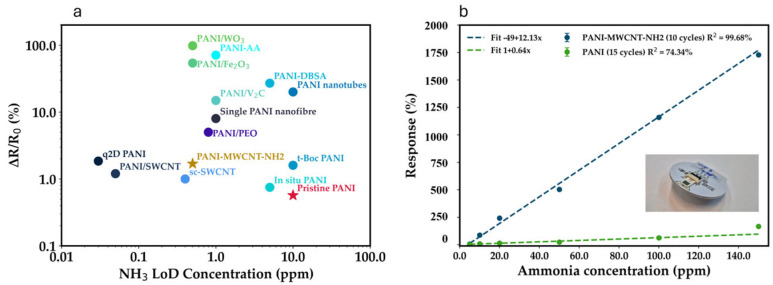
(**a**) Plot of Δ*R*/*R*_0_ vs. NH_3_ concentration of our PANI-MWCNT-NH_2_ composite (STAR symbol) in comparison to other reported PANI-based sensors from the literature. (**b**) Comparison of the responses of pristine PANI and composite PANI-MWCNT-NH_2_ sensing layers to gaseous ammonia (data from ref. [22]).

To enhance its charge transport properties and active surface area, PANI is being combined with carbon-based nanomaterials, including multi-walled carbon nanotubes (MWCNTs) functionalized with –COOH or –NH_2_ groups, as well as with oxide nanostructures (e.g., Fe_2_O_3_, WO_3_, …). Such hybrid PANI/carbon composites exhibit improved conductivity, higher density of active adsorption sites, and superior sensitivity to NH_3_ compared to pristine PANI [18,19,20,21,22], as aminated MWCNTs promote interfacial interactions with PANI and facilitate efficient modulation of the electron transport pathways upon NH_3_ exposure [23]. An overview of the various investigated PANI composites [24], in terms of their NH_3_ sensing performance, is given in Figure 1; in the same plot, the functional sensing data obtained by our PANI-MWCNTs are also reported for comparison. More details about PANI-based NH_3_ sensing performance in terms of limit of detection (LoD) and sensitivity are given in Table 1. This shows the affinity of PANI composites for ammonia detection, and the wide interest in the literature for improving the performance of such systems.

Conventional sensor characterization methodologies predominantly exploit bulk electrochemical techniques—including cyclic voltammetry (CV), electrochemical impedance spectroscopy (EIS), and macroscopic conductance measurements—to assess device performance. Although these methods yield valuable data, they are inherently limited by their spatial averaging, precluding the resolution of local electrostatic heterogeneities within the films.

Nanoscale heterogeneities in surface potential, charge distribution, and interfacial polarization significantly impact gas–solid interaction mechanisms; however, their characterization remains insufficiently addressed in current research. Electrostatic force microscopy (EFM) and Kelvin probe force microscopy (KPFM) represent suitable scanning probe methodologies that enable comprehensive mapping of nanoscale electronic and electrostatic properties. These techniques facilitate the simultaneous acquisition of high-resolution topographical information and spatially resolved surface potential mappings, and, under specific operational modes, allow direct interrogation of local charge distributions. Consequently, the utilization of EFM is essential for elucidating the intricate, spatially dependent processes underlying sensor functionality at the nanoscale [28,29,30,31,32,33,34].

The present study seeks to characterize the electrostatic properties of electrochemically synthesized polyaniline (PANI) and PANI/multi-walled carbon nanotube–NH_2_ (MWCNT–NH_2_) composite thin films. With the reproducibility and stability of the PANI–MWCNT–NH_2_ sensing architecture having been previously demonstrated under gas-phase-ammonia exposure (Figure 1b), where sensors fabricated using the same electrochemical protocol exhibited consistent response and recovery characteristics [22], the present work builds on this validated platform and focuses on elucidating the nanoscale electrostatic and electrochemical mechanisms governing the observed sensing behavior.

Employing AFM in conjunction with EFM, we establish direct correlations between nanoscale morphological features, composite aggregate architecture, and the spatial distribution of local electrostatic potential and surface charge. This investigation holds particular significance within the domain of PANI-based sensor technologies, which are garnering increasing attention due to their adjustable electrical characteristics, environmental robustness, and broad applicability in electronic devices, chemical sensing, and energy storage systems. The nanoscale electrostatic phenomena revealed by EFM are validated through CV and EIS, thereby facilitating a direct association between localized charge arrangements and overall electrochemical performance. Through the integration of functionalized MWCNT-NH_2_ within PANI matrices, we demonstrate enhancements in electrical conductivity, mechanical integrity, and sensitivity, underscoring the potential of these composites for advanced sensor applications. By spatially resolving the electrostatic landscape at the nanoscale, this work elucidates the influence of microstructural heterogeneity and nanotube-enabled percolation pathways on the functional attributes and operational efficacy of PANI-based composite sensors.

## 2. Materials and Methods

### 2.1. Reagents

Aniline monomers, sulfuric acid (H_2_SO_4_), potassium chloride (KCl), phosphate-buffered saline (PBS), potassium ferricyanide/potassium ferrocyanide redox couples ([Fe(CN)_6_]^3−^/^4−^), and other analytical-grade reagents were purchased from (Merck Group, Darmstadt, Germany). Amine-functionalized multi-wall carbon nanotubes (MWCNT–NH_2_; outer diameter, <20 nm; inside diameter, 4 nm; Ash, 0 wt%; purity, >99 wt%; length, 1–12 um) were supplied by Cheap Tubes Inc. (Cambridgeport, VT, USA). Silver conductive paste used for electrical contacts was obtained from RS Components S.r.l (Sesto San Giovanni, Italy). All aqueous solutions were prepared using ultrapure water.

### 2.2. Electrosynthesis

Polyaniline and PANI–MWCNT–NH_2_ films were electrodeposited via cyclic voltammetry (CV) following the same protocol described in our previous work [22], with an electropolymerization process consisting of 20 cycles.

### 2.3. Sensor Preparation

The sensors were deposited on miniaturized gold interdigitated electrodes (IDEs) fabricated on 0.1 mm thick alumina substrates (KYOCERA, Esslingen, Germany), following the same design and fabrication protocol as previously reported by our group [22]. The IDEs consisted of 80 electrode pairs with a digit width of 10 µm and an active area of 5 mm × 8 mm.

### 2.4. Characterizations

Electrochemical impedance spectroscopy (EIS) and cyclic voltammetry (CV) measurements were carried out using a VoltaLab PGZ100 potentiostat/galvanostat (Radiometer Analytical, France) and the VoltaMaster 4 software. Impedance spectra were recorded over a frequency range of 100 kHz to 0.1 Hz by applying a small-amplitude sinusoidal perturbation around the open-circuit potential. EIS data were fitted using a Randles-type equivalent circuit consisting of the solution resistance (Rs), charge-transfer resistance (Rct), and a constant phase element (CPE) to account for non-ideal capacitive behavior at the electrode–electrolyte interface. The same circuit configuration was applied to all impedance spectra. Curve fitting was performed using VoltaMaster 4 software (Radiometer Analytical), and fitting quality was evaluated based on the chi-square (χ^2^) values provided by the software.

AFM–EFM measurements were recorded with a PARK NX10 instrument (Park Systems, Republic of Korea) under ambient condition at room temperature using a cantilever SPARK 70 with a Pt-coated Si tip (frequency ≈ 55 kHz, Spring Constant ≈ 2 N/m, Q factor ≈ 230).

In this configuration, AFM/EFM topography and potential maps were acquired in non-contact mode, the topography being obtained through the tip scanning the surface with the cantilever oscillating in frequency. Simultaneously, an AC bias of frequency ω was applied to the tip, which senses the electrostatic force of the charged (biased) surface. The tip motion affected by the force was analyzed and yielded the EFM amplitude and phase, giving information on surface charges. Samples were biased at 3.0 V, and Ag paste was used to make the electrical contact between the sample and the metallic sample holder. Scan rates were varied in the 0.1–0.3 Hz range. AFM and EFM data were analyzed with the Smart Analysis software-Ver. 1.4.3-1480 provided by Park Instrument. AFM images were processed with the flatten function to correct sample tilting when needed.

In EFM, the fundamental operating principle relies on the detection of electrostatic forces between a conductive probe tip and a biased sample surface. When a voltage is applied between the tip and the sample, an electrostatic force (F_e_) proportional to the quantity of charge and electrical field gradients present in the sample surface arises, leading to the cantilever bending (X) [35]. Thus, by scanning the tip across the sample surface, EFM enables qualitative mapping of local charge distributions.

## 3. Results

### 3.1. AFM and EFM Nanoscale Mapping

**Figure 2:**
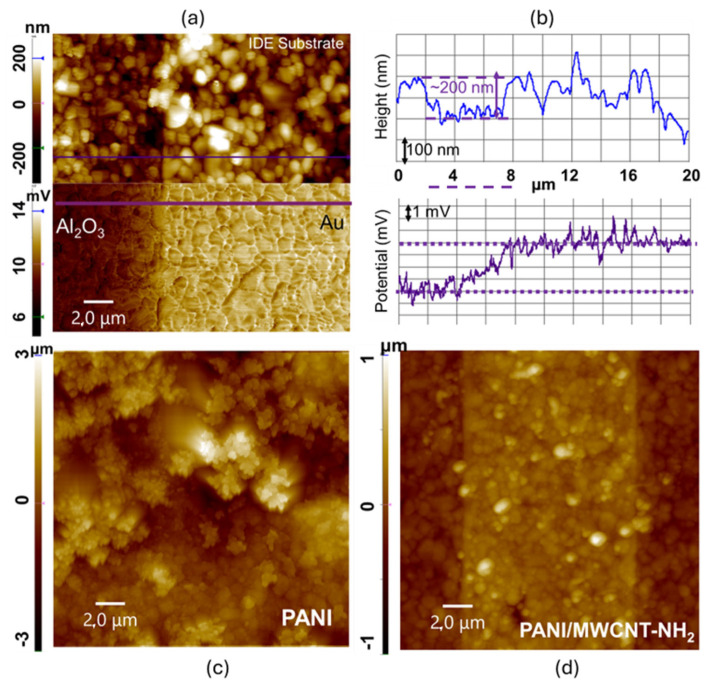
AFM topography images at a 20 µm × 20 µm scan scale of (**a**) pristine IDE substrate, with the bottom panel comprising an EFM map showing the conductive Au and insulating Al_2_O_3_ interdigit (each 10 µm wide). (**b**) The corresponding line profiles of height (top panel) and potential (bottom panel), taken along the purple lines in (**a**). (**c**) PANI electrodeposited on IDE. (**d**) PANI-MWCNT-NH2 electrodeposited on IDE (20 cycles of CV (both (**c**,**d**))).

Figure 2 shows the AFM topography of PANI-only films and of PANI/MWCNT-NH_2_ obtained by depositing PANI together with a suspension of MWCNT-NH2 after 20 CV-cycles, as well as of the starting IDE substrate. Specifically, Figure 2a shows the AFM topography and corresponding EFM potential map of the interdigitated Au/Al_2_O_3_ substrate consisting of 10-µm-wide Au electrodes with an approximate height of 200 nm, as confirmed by the topographic line profile in Figure 2b. Despite the rough morphology due to the grain structure of Au/Al_2_O_3_, a clear potential contrast—arising from the distinct electrical properties of metallic Au versus insulating Al_2_O_3_—is observed in the corresponding EFM map (Figure 2a). The measured potential difference of ~3.8 ± 0.2 mV demonstrates the sensitivity of EFM in resolving local charge distributions independently of surface roughness and morphology, consistent with previous studies highlighting EFM’s ability to discriminate conductive/insulating regions in heterogeneous thin-film systems. The PANI-only films (Figure 2c) display multilayered granular domains formed by the aggregation of individual polymer particles with a diameter < 200 nm. These grains exhibit lateral dimensions of approximately 1 µm, and typical heights in the 800 nm–1 µm range, in agreement with earlier observations of PANI emeraldine salt films, where granular and micro-/macro-aggregated morphologies are characteristic of the doped forms of PANI. AFM studies have shown that PANI undergoes significant morphological variations depending on electrochemical state, dopant type, and synthesis route, often exhibiting increased roughness in conductive vs. nonconductive states. With increasing film thickness, these PANI grains coalesce into larger macroaggregates, reflecting the intrinsic tendency of PANI chains to form hierarchical microstructures [31]. Figure 2d highlights the significant morphological differences introduced by incorporating NH_2_-functionalized MWCNTs into the PANI matrix. Although PANI-only films deposit uniformly over both Au and Al_2_O_3_ at a 20 µm × 20 µm scan scale, the PANI/MWCNT-NH_2_ composite deposits predominantly on Au electrodes. Thus, a visible contrast due to the Au and Al_2_O_3_ of the IDEs can again be seen. From this preferential localization of deposition, it can be inferred that the enhanced conductivity introduced by MWCNTs directs the electrodeposition along conductive Au electrode pathways.

This phenomenon can be attributed to the formation of an interconnected nanotube-mediated conductive network, which facilitates charge transport during polymer growth. Functionalization of MWCNTs with amino (–NH_2_) groups further promotes chemical interactions with PANI. NH_2_ groups increase local electron density and create favorable hydrogen bonding sites, strengthening the PANI–MWCNT interaction. Density Functional Theory (DFT) and molecular dynamics simulations have also demonstrated that NH_2_-functionalized PANI and CNTs exhibit enhanced adsorption affinity for ammonia (NH_3_) due to acid–base interactions, increased local charge density, and stronger non-covalent bonding—effects that also influence electrodeposition behavior by locally enriching NH_3_ and modifying doping levels [36]. During electrodeposition, PANI macromolecules are known to wrap around MWCNTs via π–π stacking and electrostatic interactions, increasing film connectivity and electroactive surface area.

**Figure 3:**
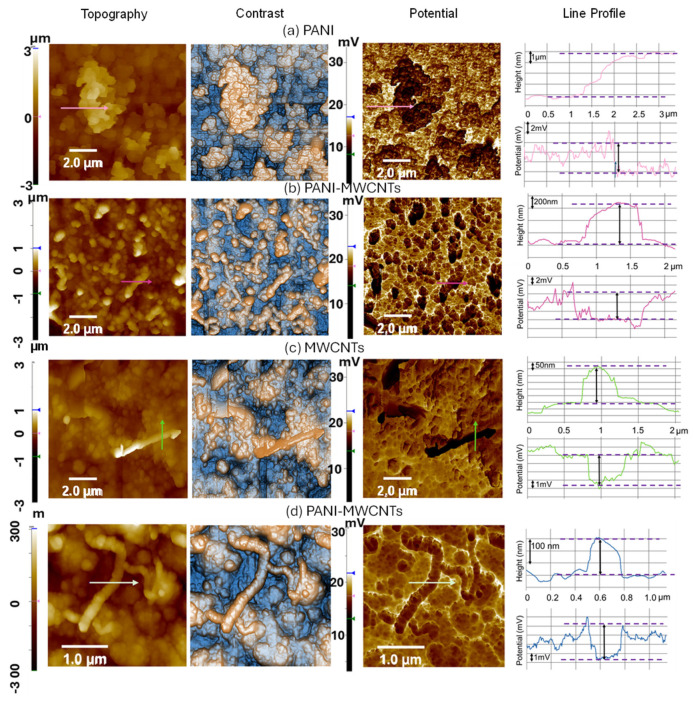
Images of AFM topography at a 10 µm × 10 µm scan scale (left panel), enhanced-palette mode contrast topography (central panel—yellow-blue maps), and EFM maps (right panel) with the corresponding line profiles of (**a**) the PANI sample, (**b**) PANI/MWCNT-NH_2_ with different MWCNTs, (**c**) another zone of the PANI/MWCNT sample emphasizing the presence of an MWCNT spotted on the polymer surface, and (**d**) an MWCNT embedded in a polymer layer. All samples are electrodeposited with 20 cycles.

Figure 3 provides better insight into the PANI-MWCNT films with EFM potential maps that spatially resolve local charge distribution within the examined thin films. The topography of PANI in Figure 3a shows large macro-aggregates. The corresponding observed enhancement in charge density within the darker domains of PANI macro-aggregates (see the EFM map in Figure 3a) is ascribed to the presence of larger polymeric assemblies, which promote augmented charge trapping and accumulation, consistent with the established literature on charge localization in conducting polymer systems. It is inferred that accumulation of charge in those macro-aggregates of PANI causes significant imbalance in charge distribution and, consequently, in conductivity, which may affect the sensing response to NH_3_. Conversely, Figure 3b–d demonstrate that the addition of MWCNTs as dopants substantially influences the topography and charge density distribution within the PANI matrix. AFM topography and contrast images of PANI-MWCNTs in Figure 3b–d reveal characteristic “worm-like” or fibrillar aggregates corresponding to MWCNTs coated with PANI chains. As an example, Figure 3c clearly shows a worm-like aggregate approximately 4 μm in length and ~500 nm in diameter that originates from an MWCNT-NH_2_ enveloped by PANI. The corresponding EFM map and amplitude profile reveal that these PANI-coated MWCNT structures exhibit higher electrical conductivity than the surrounding PANI matrix, with elevated local potentials ranging from 4.6 to 6.4 mV, indicative of enhanced charge transport. Moreover, the analysis of Figure 3b suggests that MWCNTs play a pivotal role in suppressing the formation of large PANI aggregates and instead facilitate the creation of a more homogeneous, percolative network of conductive pathways mediated by PANI-wrapped MWCNTs. This synergy between PANI and functionalized CNTs is well documented and includes improvements in dielectric performance, optical properties, and electrochemical responses. The worm-like nanotube–polymer aggregates and the markedly different microstructure of the composite film compared to pristine PANI confirm that MWCNTs—especially NH_2_-functionalized ones—act not only as conductive scaffolds but also as chemical modulators of PANI electrodeposition, morphology, and local dopant dynamics. This MWCNT doping methodology enables controlled modulation of the film’s electronic properties, thereby enhancing its electrical conductivity—a feature critical for advanced electronic and sensor applications. Recent studies corroborate that MWCNT incorporation improves charge carrier mobility and network connectivity, leading to superior conductive pathways [37,38,39].

### 3.2. Electrochemical Validation of AFM and EFM Observations

#### 3.2.1. Baseline Electrochemical Behavior and Electroactive Area

**Figure 4:**
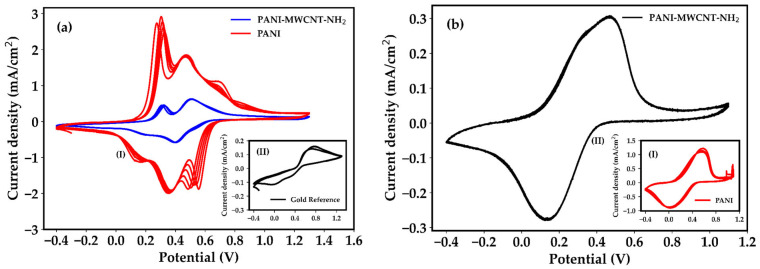
(**a**) Cyclic voltammograms of pristine PANI (red) and PANI–MWCNT–NH_2_ composite (blue) recorded on interdigitated gold electrodes in 0.1 M H_2_SO_4_ containing the ferri/ferrocyanide redox couple (I); the cyclic voltammogram of the bare interdigitated gold electrode recorded in the same electrolyte is shown in the inset (II). (**b**) Cyclic voltammograms of pristine PANI (I, inset) and PANI–MWCNT–NH_2_ composite (II) recorded in 0.1 M KCl, highlighting the intrinsic electrochemical activity of the polymer-based films in the absence of an external redox probe.

The interfacial electron transfer properties of the electrodeposited films were first evaluated using the reversible [Fe(CN)_6_]^3−^/^4−^ redox couple in 0.1 M H_2_SO_4_. A bare interdigitated gold electrode was used as reference to decouple the contribution of the conductive substrate from that of the polymer-based active layers. Cyclic voltammetry revealed a progressive increase in faradaic currents when moving from the bare Au IDE to PANI and further to the composite-modified electrodes (Figure 4a, panels I and II). The higher peak currents observed in pristine PANI (Figure 4a, panel I, red curve), compared to both the bare Au interdigitated electrode (panel II) and the PANI–MWCNT–NH_2_ composite (Figure 4a, panel I, blue curve), indicate a larger electroactive polymer fraction, which is consistent with the highly developed surface morphology evidenced by AFM. AFM analysis further revealed that PANI forms compact aggregates and exhibits localized charge distribution, with electronic transport predominantly governed by hopping processes between polymer domains. More importantly at this stage, the magnitude of the faradaic current measured by cyclic voltammetry mainly reflects the electroactive area accessible to the redox probe rather than the intrinsic electron transfer kinetics of the films.

#### 3.2.2. Intrinsic Electrochemical Activity of PANI and Composite Films

To probe the intrinsic electrochemical activity of the polymer matrix (which reflects the material’s intrinsic capability to engage in electrochemical processes, irrespective of external redox probes, substrate effects, or electrode geometry), CV was subsequently performed in 0.1 M KCl in the absence of any redox mediator. Under these conditions, the voltametric response originates from the redox transitions of polyaniline itself, and involves proton-coupled electron transfer between its leucoemeraldine, emeraldine, and pernigraniline states. As shown in Figure 4b, pristine PANI exhibits higher faradaic peak currents but a less regular voltametric response, whereas the composite displays lower peak currents accompanied by more gradual and stabilized behavior. Notably, the peak-to-peak potential separation (ΔE_p_) is smaller for the PANI–MWCNT–NH_2_ (ΔE_p_ = 316 mV) composite than for pristine PANI (ΔE_p_ = 579 mV), indicating faster electron transfer kinetics at the electrode–electrolyte interface. Those observations suggest that, although the polymer-only film contains a larger fraction of redox-active material, the incorporation of MWCNT–NH_2_ enhances charge propagation and reduces kinetic limitations by providing efficient conductive pathways within the composite matrix. Such behavior is consistent with the AFM and EFM observations, which revealed an interconnected worm-like morphology and a more delocalized charge distribution in the composite, characteristics that favor efficient charge transport across the active layer.

### 3.3. Charge-Transfer Kinetics and Correlation with EFM Charge Distribution

**Figure 5:**
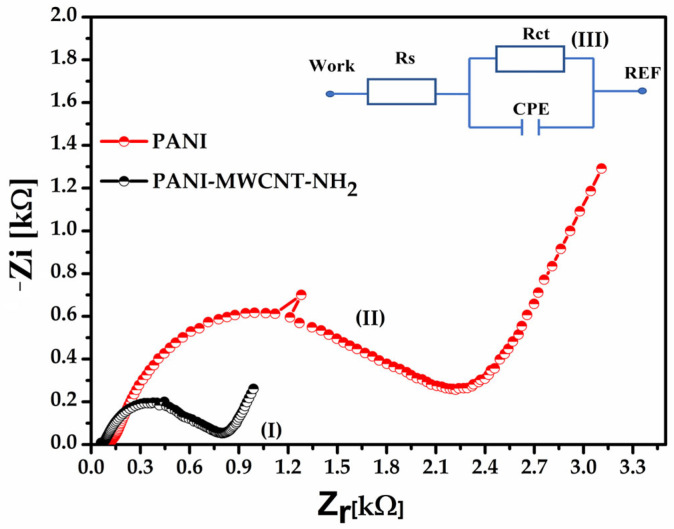
Nyquist plots (−Zi vs. Zr) of pristine PANI (red symbols) and PANI–MWCNT–NH_2_ composite (black symbols) recorded on interdigitated gold electrodes in 0.1 M KCl, highlighting the intrinsic interfacial charge-transfer behavior of the two active layers in the absence of an external redox probe. The inset (III) presents the modified Randles equivalent circuit used for fitting, which comprised the solution resistance (Rs) in series with a parallel Rct–CPE element. Rs represents the electrolyte resistance, Rct corresponds to the interfacial charge-transfer resistance, and the constant phase element (CPE) accounts for the non-ideal capacitive response arising from surface roughness and heterogeneity of the polymer/composite films.

While cyclic voltammetry provides qualitative insight into electroactive surface area and intrinsic redox activity, electrochemical impedance spectroscopy (EIS), represented as Nyquist plots (−Z″ vs. Z′), provides quantitative information on interfacial charge-transfer kinetics. Quantitatively, this structural and electrostatic reorganization is reflected in a marked reduction in the charge-transfer resistance (Rct), which decreases from an average of 1.2 kΩ for pristine PANI (Figure 5, I) to 400 Ω for the PANI–MWCNT–NH_2_ composite (Figure 5, II). This nearly threefold decrease in Rct confirms that MWCNT incorporation enhances interfacial electron transfer efficiency rather than merely increasing the electroactive area. The Rct values were obtained by fitting the impedance spectra using the VoltaMaster 4 software and a modified Randles equivalent circuit. The fitting quality was verified through low χ^2^ values and randomly distributed residuals, which confirmed the robustness of the extracted parameters. This behavior correlates directly with the EFM observations, where larger ΔE_p_ values for PANI indicated more localized charge states, whereas the reduced electrostatic contrast observed for the composite reflects charge delocalization. The lower Rct values measured for the PANI–MWCNT–NH_2_ films therefore support the formation of nanotube-mediated percolation pathways that facilitate long-range electron transport across the interface. These results demonstrate that NH_2_-functionalized MWCNTs enhance baseline electron transfer efficiency by promoting charge delocalization and establishing percolative transport pathways across the composite film.

### 3.4. Ammonia-Induced De-Doping: Comparative CV Response

**Figure 6:**
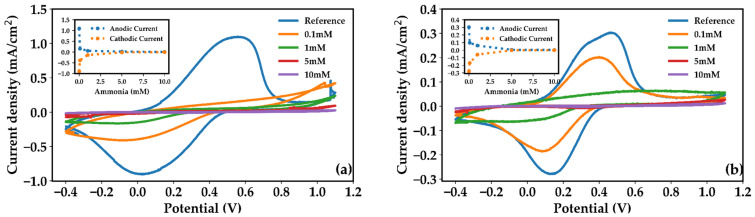
(**a**) Cyclic voltammograms of pristine PANI and PANI–MWCNT–NH_2_ composite recorded on interdigitated gold electrodes in 0.1 M KCl in the absence (reference) and presence of increasing concentrations of dissolved ammonia (0.1, 1, 5, and 10 mM). (**b**) Cyclic voltammograms of the PANI–MWCNT–NH_2_ composite recorded under the same experimental conditions, highlighting the progressive modulation of the voltametric response upon increasing the NH_3_ concentration. All measurements were performed at a scan rate of 50 mV s^−1^.

The interaction with dissolved ammonia was investigated by recording cyclic voltammograms in 0.1 M KCl containing increasing concentrations of dissolved NH_3_. For pristine PANI, exposure to dissolved ammonia induces a rapid and strongly nonlinear suppression of the voltametric response (Figure 6a), consistent with fast deprotonation of the emeraldine salt and abrupt loss of mobile charge carriers. In contrast, the composite (Figure 6b) exhibits a more progressive and controlled evolution of the CV signal with increasing ammonia concentration. This behavior suggests that, although ammonia effectively interacts with the active layer in both cases, the nanotube network redistributes the de-doping-induced perturbation through alternative charge transport pathways, leading to a more progressive modulation of the electrochemical response.

While cyclic voltammetry mainly reflects the accessible electroactive polymer fraction, electrochemical impedance spectroscopy provides a quantitative assessment of the interfacial charge-transfer kinetics, allowing the distinct contributions of PANI and MWCNTs to be disentangled.

### 3.5. Impedance Response Under Ammonia Exposure

**Figure 7:**
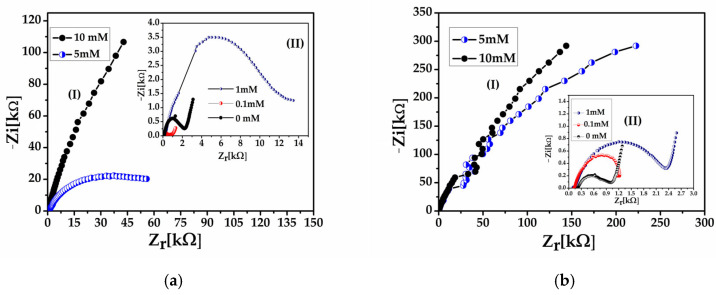
Nyquist plots of (**a**) pristine PANI and (**b**) PANI–MWCNT–NH_2_ composite recorded on interdigitated gold electrodes in 0.1 M KCl after exposure to increasing concentrations of dissolved ammonia. In each panel, (I) shows the impedance response at higher ammonia concentrations (5 and 10 mM), highlighting the pronounced increase in charge-transfer resistance, while (II) presents the corresponding low-concentration regime (0, 0.1, and 1 mM) for clarity. The evolution of the impedance response and effective semicircle diameter reflects the progressive modulation of interfacial charge-transfer processes induced by ammonia adsorption. Fitting was performed using the equivalent circuit presented in Figure 5.

The impact of ammonia on electron transfer kinetics was further elucidated by EIS measurements performed after exposure to increasing NH_3_ concentrations. As shown in Figure 7a, pristine PANI exhibits a rapid and strongly nonlinear increase in charge-transfer resistance and reaches values on the order of hundreds of kilo-ohms (≈661.7 kΩ) at the highest ammonia concentration (panel I), indicative of fast and poorly controlled de-doping of the polymer matrix. At lower ammonia concentrations (panel II), the impedance response of PANI is weakly defined, with no clearly developed semicircular feature, suggesting limited accommodation of NH_3_ at the polymer–electrolyte interface.

In contrast, the PANI/MWCNT–NH_2_ composite (Figure 7b) displays a much more pronounced and concentration-dependent increase in *R_CT_*, approaching the mega-ohm range (≈1.051 MΩ) at 10 mM NH_3_ (panel I). At low ammonia concentrations (panel II), a partially developed semicircular response is still observed, indicating a more organized interfacial charge-transfer process prior to extensive de-doping. This amplified impedance response reflects enhanced ammonia interaction and preconcentration within the nanotube-containing architecture, leading to a more extensive modulation of the interfacial electron transfer process. A clear difference in the ammonia interaction mechanism thus emerges from the impedance data. While the PANI–MWCNT–NH_2_ composite exhibits two-regime behavior—characterized by gradual adsorption at low concentrations (Figure 7b, panel II) followed by a threshold-driven response at higher ammonia levels (Figure 7b, panel I)—pristine PANI responds in an abrupt and highly nonlinear manner (Figure 7a), with no evident accommodation regime. Notably, the transition from a partially defined semicircular response at low ammonia concentrations to diffusion-dominated impedance behavior at higher concentrations further supports the role of the nanotube framework in stabilizing charge transport prior to extensive polymer de-doping.

It is worth noting that the superior ammonia sensitivity of the PANI–MWCNT–NH_2_ composite compared to pristine PANI has already been demonstrated under gas phase conditions in our earlier work, where direct gas–solid interactions and efficient nanotube-assisted adsorption played a dominant role [22]. In contrast, the liquid phase electrochemical measurements discussed here display a buffered and diffusion-limited response yet remain fully consistent with the gas phase findings by highlighting the role of nanotube-mediated charge transport and response stabilization. It is well established that molecular diffusivity in gases is several orders of magnitude higher than in liquids. Typical diffusion coefficients for small gaseous species, including ammonia in air, are on the order of 10^−5^ m^2^/s, whereas analogous diffusion of solutes in aqueous media occurs with coefficients on the order of 10^−9^–10^−10^ m^2^/s, reflecting much faster mass transport in the gas phase compared to the liquid phase [40].

Under ammonia exposure, the composite exhibits a larger R_ct_ modulation compared to pristine PANI, indicating that the nanotube-mediated network does not prevent de-doping but amplifies the interfacial perturbation induced by NH_3_ adsorption.

## 4. Conclusions

This study demonstrates that the sensing behavior of PANI-based thin films is governed by the organization and transport dynamics of charge within the films. AFM and EFM reveal that the composite architecture promotes charge delocalization and the formation of conductive pathways, while CV and EIS provide functional validation of these nanoscale features. Pristine PANI, despite exhibiting a larger electroactive fraction and higher baseline faradaic currents, displays pronounced charge localization and limited electron transfer efficiency, which make its conductivity highly vulnerable to abrupt ammonia-induced de-doping. In contrast, the incorporation of NH_2_-functionalized MWCNTs generates interconnected, worm-like percolative pathways that promote charge delocalization, reduce charge-transfer resistance, and modulate electrochemical response under increasing NH_3_ concentrations. Quantitatively, MWCNT incorporation reduces ΔE_p_ from 579 mV to 316 mV and decreases Rct from ~1.2 kΩ to ~400 Ω under baseline conditions. Upon exposure to 10 mM NH_3_, Rct increases to approximately 661 kΩ for pristine PANI and to 1.05 MΩ for the PANI–MWCNT–NH_2_ composite, reflecting ~1.6-fold higher Rct values in the composite system. The incorporation of amine-functionalized carbon nanotubes promotes charge delocalization and transport resilience, leading to a gradual and threshold-controlled impedance response, and enabling the production of robust, next-generation ammonia sensors suitable for low-power environmental and biomedical applications.

## Figures and Tables

**Table 1:** Representative PANI-based ammonia sensors’ properties according to the literature.

Sensing Layer	LoD Concentration (ppm)	Sensitivity (%)
q2D PANI ^1^	0.03	1.85
PANI nanotubes ^1^	10	20
t-Boc PANI ^1^	10	1.6
PANI-DBSA ^1^	5	27
In situ PANI ^1^	5	0.75
PANI-AA ^1^	1	71
Single PANI nanofiber ^1^	1	8
PANI/PEO ^1^	0.8	5
sc-SWCNT ^1^	0.4	88
PANI/SWCNT ^1^	0.05	1.2
PANI/WO_3_ ^2^	0.5	98
PANI/Fe_2_O_3_ ^3^	0.5	54
PANI/V_2_C ^4^	1	14.9
PANI ^5^	10	0.5
PANI-MWCNT-NH_2_ ^5^	0.57	1.69

^1^ Ref. [24], ^2^ Ref. [25], ^3^ Ref. [26], ^4^ Ref. [27], ^5^ Ref. [22].

## Data Availability

Research data will be made available upon request from the corresponding author.

## Abbreviations

The following abbreviations are used in this manuscript:

PANI	Polyaniline
MWCNTs	Multi-Wall Carbon Nanotubes
PANI/MWCNT–NH_2_	Polyaniline/Aminated Multi-Wall Carbon Nanotubes
CNTs	Carbon Nanotubes
AFM	Atomic Force Microscopy
EFM	Electrostatic Force Microscopy
EIS	Electrochemical Impedance Spectroscopy
CV	Cyclic Voltammetry
DFT	Density Functional Theory
